# Biomaterial Engineering for Controlling Pluripotent Stem Cell Fate

**DOI:** 10.1155/2018/9068203

**Published:** 2018-12-05

**Authors:** Taylor B. Bertucci, Guohao Dai

**Affiliations:** Department of Bioengineering, Northeastern University, Boston, MA 02115, USA

## Abstract

Pluripotent stem cells (PSCs) represent an exciting cell source for tissue engineering and regenerative medicine due to their self-renewal and differentiation capacities. The majority of current PSC protocols rely on 2D cultures and soluble factors to guide differentiation; however, many other environmental signals are beginning to be explored using biomaterial platforms. Biomaterials offer new opportunities to engineer the stem cell niches and 3D environments for exploring biophysical and immobilized signaling cues to further our control over stem cell fate. Here, we review the biomaterial platforms that have been engineered to control PSC fate. We explore how altering immobilized biochemical cues and biophysical cues such as dimensionality, stiffness, and topography can enhance our control over stem cell fates. Finally, we highlight biomaterial culture systems that assist in the translation of PSC technologies for clinical applications.

## 1. Introduction

Pluripotent stem cells (PSCs), including embryonic stem cells (ESCs), have unique properties of self-renewal and differentiation capacities of all cell types in the body. Recent advances in induced pluripotent stem cell (iPSC) technology have implications for clinical applications including cell therapies, tissue engineering, drug screening, and *in vitro* tissue models. iPSCs come from terminally differentiated adult cells reprogrammed into a pluripotent state and therefore can be obtained directly from patients. As such, iPSCs can overcome certain complications such as immune transplant rejection, a major concern in cell therapies and tissue-engineered constructs. Furthermore, iPSC technology permits development of personalized *in vitro* models for disease susceptibility and drug response studies.

To date, the majority of *in vitro* PSC protocols depend on two-dimensional (2D) culture systems and soluble factors to control differentiation. These schemes have successfully generated cell types from all three germ layers; however, there are still major limitations that must be addressed. First, these methods cannot fully recapitulate native 3D environments. We know cell-cell and cell-matrix interactions play critical roles in development and tissue maturation. Additionally, 2D differentiation systems are severely limited in clinical translation due to the lack of scalability. Biomaterials offer an alternative approach that may overcome these limitations.

**Figure 1:**
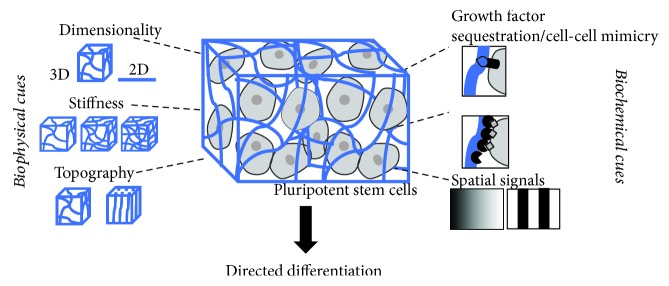
Biomaterial characteristics that are employed to influence PSC fate and their potential therapeutic applications.

Here, we review how biomaterials have been designed and created to control PSC fate. First, we explore how researchers have tailored the biophysical and biochemical characteristics of biomaterials to direct differentiation or maintain pluripotent states.  Figure 1 summarizes different characteristics of biomaterials that can be manipulated to instruct PSC fate. Finally, we summarize how biomaterial approaches can address hurdles for translating stem cell technologies into clinically viable therapies. Overall, PSC technology has great potential in advancing personalized medicine, and biomaterial engineering is a powerful tool for accelerating successful implementations of PSC technology.

## 2. Scaffolds for Pluripotent Stem Cell Cultures

There are important design features to consider when engineering a scaffold for stem cell cultures. Minimally, scaffolds must support stem cell survival. Native extracellular matrix (ECM) proteins, such as Matrigel or collagen, are commonly used to create microenvironments because they readily mimic *in vivo* surroundings, containing motifs that support cell attachment and growth. When creating scaffolds for directed differentiation, researchers are often motivated to create a scaffold that mimics the tissue composition of the desired cell type. For example, biomaterials for osteogenic biomaterials have been synthesized using hydroxyapatite [1], an inorganic mineral unique to bone tissue. Similarly, neurogenic scaffolds often contain hyaluronic acid [2, 3], an abundant glycosaminoglycan found in brain ECM.

Other approaches include using synthetic polymers to create scaffolds for PSC. Synthetic scaffolds can create a bioinert base structure to build up from. Biocompatible polymers are commonly employed for these scaffolds, such as polycaprolactone (PCL) [4–6], poly(lactic-co-glycolic acid) (PLGA) [7–10], and poly(ethylene glycol) (PEG) hydrogels [11–15]. Synthetic scaffolds often require additional protein or peptides to improve cell attachment and matrix degradation. Researchers sometimes choose to not use the full ECM protein within their designs since they can be expensive and more sensitive to material processing techniques. Instead, bioactive peptides that contain the active sites of the protein are engineered as an easily manipulated economic alternative. For example, fibronectin's binding sequence is comprised of three amino acids: arginylglycylaspartic acid (RGD) [3, 14, 16–20]. This simple peptide is routinely incorporated into scaffold designs to support cell attachment. Another example is the use of matrix metalloproteinase (MMP) peptides. MMPs are enzymes that are responsible for degrading ECM targeted by a specific amino acid sequence. Synthetic biomaterials can be engineered to enzymatically degrade based on the concentrations of MMP degrading peptides incorporated within the network [14].  Table 1 provides an overview of commonly employed natural and synthetic biomaterials for PSC culture systems.  Table 2 summarizes common ECM-inspired bioactive molecules that are included within scaffold designs via immobilization chemistry to support stem cell cultures. Overall, there is an assortment of successful scaffold designs that can support PSC cultures and has been used for deriving a variety of cell fates.

## 3. Biophysical Cues that Influence PSC Fate

Biophysical cues can be varied to influence PSC fate. In primary cell cultures, biophysical environmental cues are known to modulate cell attachment, spreading, and migration. Here, we will explore how dimensionality, stiffness, and topography are used to manipulate PSC fate.

### 3.1. Dimensionality

3D biomaterial platforms exhibit enhanced PSC differentiation efficiency compared to 2D cultures [40, 46–48]. First, in the absence of soluble factors, 3D microenvironments alone promote differentiation along certain PSC fates [22, 49]. For example, PSC differentiation performed within a 3D biomaterial enhanced an osteogenic commitment compared to 2D cultures [22]. Other differentiation schemes are enhanced in 3D cultures but require soluble factors to improve efficiency in PSC differentiation. For example, Baharvand et al. report earlier a higher expression of hepatocyte genes 3D differentiation compared to 2D [48].

Functionality of the terminally differentiated cell population is also effected by the dimensionality of the differentiation protocol [11, 15, 40, 47]. For example, PSC-derived endothelial cells exhibited different global gene expression profiles depending on if the endothelial cells were differentiated in 2D or 3D cultures [15]. In neural differentiation, researchers observed certain genes more strongly expressed in 3D-derived neurons compared to 2D [11]. In particular, they reported a 30-fold increase in viability of midbrain dopaminergic neurons when differentiated within a 3D biomaterial platform. Moreover, their electrophysiological characteristics were also superior to populations obtained from 2D differentiation.

Dimensionality also plays an interesting role with respect to PSC response to biochemical cues. Heydarkhan-Hagvall et al. found that ECM molecules induced endothelial differentiation differently when PSCs are cultured in 2D or 3D [50]. Specifically, they reported collagen IV coating induced vascular differentiation best in 2D while vitronectin performed best in 3D [50]. For another example, Puig-Sanvicens et al. reported cardiomyocyte differentiation within a 3D self-assembling peptide hydrogel did not require the addition of ascorbic acid, whereas cardiomyocyte differentiation in 2D did require ascorbic acid [37]. This highlights the interplay between biophysical environmental cues and biochemical signals and the importance of addressing both when engineering a system for PSC differentiation.

### 3.2. Stiffness

**Figure 2:**
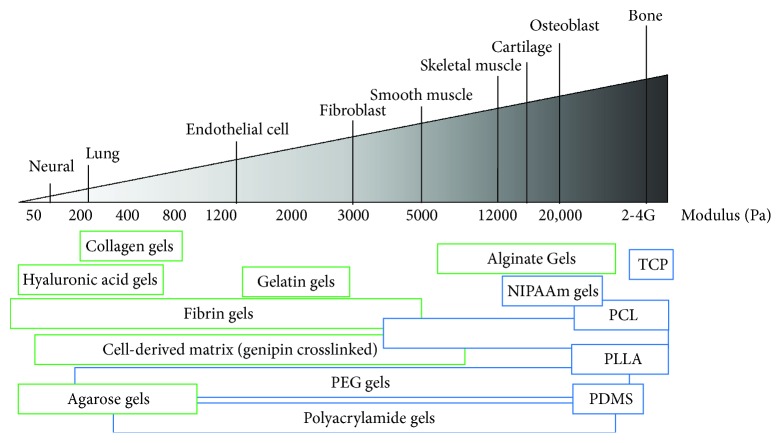
Tissue stiffness *in vivo*, adopted figure from Butcher et al. [51], and the range of stiffness that can be achieved by natural (green) and synthetic (blue) biomaterials [25, 31, 52–70]. Stiffness ranges illustrate stiffness values reported in the literature, not necessarily the complete stiffness range attainable of these materials.

Stiffness, a physical characteristic of materials, can influence cell behavior such as motility, morphology, and in the context of stem cells, cell fate. Tissue culture plastic (TCP) substrates are routinely used for stem cell culture. However, the stiffness of TCP is several orders of magnitude stiffer than the stiffness PSCs are exposed to in the body. Biomaterials are an ideal platform to evaluate the effects of stiffness on stem cell fate. Common methods for manipulating stiffness include adjusting the precursor monomer concentration and/or changing the concentration of the crosslinking agent.  Figure 2 illustrates the range of tissue stiffness found in the body and highlights stiffness ranges of commonly employed biomaterials.

Studies have demonstrated that stiffness is an important design feature to consider in stem cell maintenance culture [29, 31, 58, 71], iPSC reprogramming [66], and differentiation strategies [25, 29, 58–60, 67, 72]. One study suggests stiffness is a major factor in maintaining long-term pluripotency (source). Specifically, PSCs cultured on cell-derived matrix (200 Pa) for 35 days retained more than 90% Oct4+ PSCs compared to approximately 80% in cultures performed with stiffness of 100 Pa [31]. However, a different study indicated stiffer substrates (~2.5 MPa) promote pluripotency [58]. Within the field, there are inconsistent conclusions about what stiffness levels are optimal for pluripotent maintenance. The various PSC lines, maintenance media formulation, and substrate composition may be contributing to the different observations between publications.

Stiffness has been widely studied in the context of promoting germ layer commitment. Jaramillo et al. achieved fine-tune stiffness from 4 to 247 Pa with fibrin gels and concluded endoderm commitment was stronger when cultured within softer substrates compared to other tested stiffness in both 2D and 3D [25]. Richardson et al. used stiffness ranging from 7 to 90 KPa for 3D pancreatic differentiation and observed that while stiffer alginate capsules enhanced definitive endoderm, they later suppressed pancreatic progenitor induction [72]. For mesoderm lineage differentiation studies, results showed mesodermal Brachyury expression was upregulated as a response to increased substrate stiffness, with maximum expression levels measured within the MPa stiffness range [59]. In a separate study, the greatest mesodermal gene expression was on PDMS stiffness at 1.7 MPa compared to PDMS at 3 KPa and TCP [60]. This study illustrated how stiffness alone, not including any biochemical cues, can be used to activate Wnt signaling, a critical pathway in mesoderm commitment. These studies both suggest that a stiffer substrate (~MPa) is most appropriate for guiding PSCs towards mesodermal lineage stem cell fates. Finally, specific stiffness has shown to be effective for ectoderm and, furthermore, neural differentiation. Soft substrates (~100 Pa) promoted earlier neural ectoderm commitment as well as an increase in total neurons and dopaminergic neurons compared to differentiation on stiffer gels (KPa) or TCP [67].

Overall, biomaterials are readily available and easily configurable to modulate stiffness and enhance different PSC fates. Precise stiffness levels optimal for pluripotency and different PSC fates remain unclear, and further exploration is needed.  Table 3 summarizes studies that investigate stiffness effects on PSCs using biomaterials.

### 3.3. Topography

**Figure 3:**
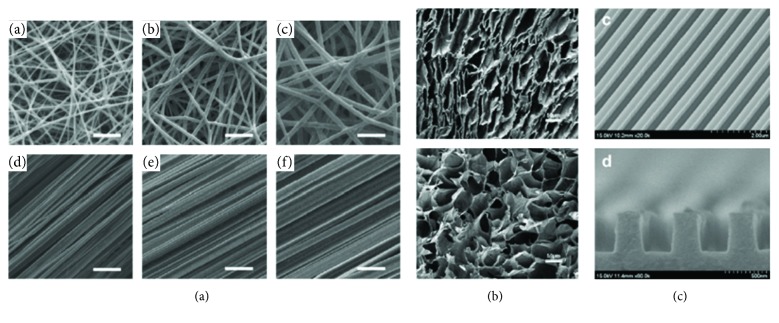
SEM images of different topographies used to influence PSC fate. (a) Electrospun meshes with differently sized fiber diameters, in random or aligned fiber orientation, investigated with respect to germ layer commitment [73]. (b) Rod (top) or sphere (bottom) pore-shaped scaffold used for bone differentiation. Study found spherical pores supported osteogenic fate better than rod shapes [1]. (c) Nanosize surface grooves instructed PSCS into neuronal lineage without additional inducing agents [78].

Topography can play an active role in manipulating PSC fate. Researchers have developed materials with different topographical cues for PSCs through a variety of methods including electrospinning [4–9, 73–75], pore shape manipulation [1, 76], and surface treatments [77–79]. Examples of topographies obtained via these types of processing are illustrated in Figure 3.

Investigation into topographical cues as potent PSC fate regulators is focused within three major differentiation lineages: chondrogenic [9, 74], osteogenic [1, 6, 28, 73, 76], and neural [4, 5, 7, 8, 73, 78]. Many studies have reported that fibrous, electrospun microenvironments favor chondrogenic [9, 74] and neural [4, 5, 8] differentiations. Specifically, aligned fibers enhanced tendon-specific genetic profiles while suppressing osteogenic differentiation when compared to randomly aligned fibers [74]. Electrospinning techniques can be used to alter the diameter of the fibers. Fiber diameter has been reported to enhance different PSC fates. For example, Cooper et al. investigated how aligned, random fiber substrates with varying fiber diameter affected PSC commitment to ectoderm, mesoderm, and endoderm lineages (Figure 3(a)). In this study, neural markers (ectoderm) were enhanced on aligned fibers with larger size diameter fibers (400 nm), osteogenic markers (mesoderm) were promoted on either random or aligned fibers with smaller diameter fibers (200 nm), and hepatic markers (endoderm) were promoted on randomly aligned 200 nm fiber substrate [73]. Osteogenic differentiation was shown to respond to pore shape within a scaffold (Figure 3(b)). In particular, sphere-shaped pores supported PSC osteogenic fate compared to pores with a rod shape. Finally, surface nanosized features are another form of topography shown to promote pluripotent maintenance [77, 79] and neural differentiation [78] (Figure 3(c)). Ryul et al. showed that topographical cues alone promoted neuronal differentiation without the need of biochemical cues, and moreover, they concluded this topography can be used for rapid and efficient guidance of neuronal PSC fate.

## 4. Immobilized Biochemical Cues that Influence PSC Fate

Another way biomaterials can improve control of PSC differentiation is through the immobilization of biochemical signals. Current practices rely on soluble factors to influence stem cells *in vitro*; however, this method is unable to capture all signaling that occurs *in vivo*. There is a large cohort of signals that are immobilized natively through matrix sequestration and cell-cell interactions. Here, we will summarize common immobilization chemistries used to tether proteins and peptides to biomaterials and explore how these immobilized signals are used to mimic growth factor sequestration or multivalent signaling to control PSC fate. Additionally, we will address how specific spatial biochemical patterns are used as a tool for studying PSC differentiation.

### 4.1. Covalent Bioconjugation Strategies

**Figure 4:**
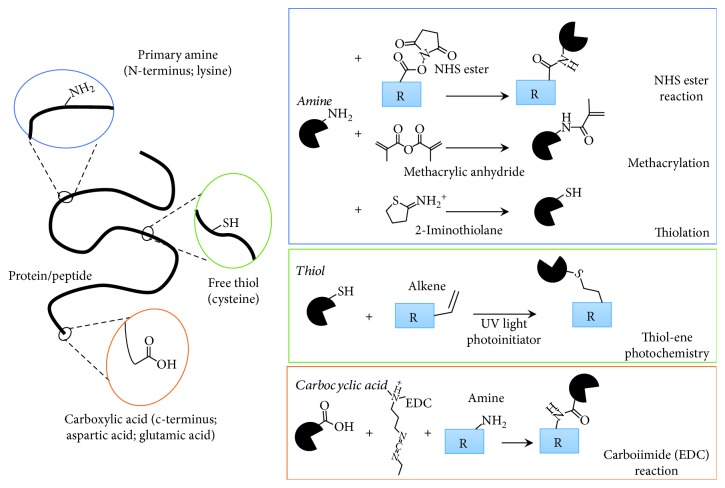
Commonly targeted modification sites on proteins/peptides and their respective covalent bioconjugation chemistries.

There are a variety of chemical reactions used to immobilize proteins/peptides to biomaterials. Certain amino acid side groups are readily available for modification including lysine, cysteine, aspartic acid, and glutamic acid.  Figure 4 illustrates targeted motifs and their associated bioconjugation modification strategies.

Primary amines, located on the lysine amino acid side group or the N-terminus of polypeptides, are common targets for bioconjugation. Amines can react with esters or anhydrides to form amide bonds. One strategy includes polymer linkers with one end modified with n-hydroxysuccinimide (NHS) ester to react with the protein and the other end functionalized with a scaffold reactive group. Another method is to react proteins with methacrylic anhydride. This converts primary amines directly into methacrylate groups for subsequent photochemistry, seen commonly with gelatin methacrylate (GelMA) and hyaluronic acid methacrylate (HAMA) scaffolds [3, 20, 22]. Lastly, amine conjugation is also used with polydopamine-coated substrates for surface immobilization of peptides/proteins [18, 19, 58].

Another target for bioconjugation is thiol motifs located on cysteine amino acids. Proteins generally require a modification to add a free thiol for conjugation since native cysteines are typically involved in the protein structure via disulfide bonding. Primary amines on the protein surface can be readily converted into a thiol. For peptides, an additional cysteine can be added during peptide synthesis to provide the bioconjugation target. Thiol-ene is a popular photochemistry approach for scaffold crosslinking and protein/peptide conjugation. This step-growth reaction is often referred to as “click chemistry” due to its fast speed and high efficiency [12, 16].

Carboxylic acids, located on side groups of aspartic acid, glutamic acid, and the c-terminus of polypeptides are readily available motifs for carbodiimide (EDC) chemistry and are a common approach for creating biomaterials for PSCs [24, 34, 35, 43, 45, 80–84]. Similar to amine-targeted approaches, this motif can be used to introduce heterobifunctional linkers or directly used to covalently conjugate proteins/peptides with biomaterial scaffolds. Conclusion sentence?

### 4.2. Immobilized Biochemical Cues for Mimicking Growth Factor Sequestration

Protein immobilization to biomaterial scaffolds is aimed at better recapitulating native ECM growth factor sequestration. Heparin is a glycosaminoglycan that captures a variety of growth factors within the ECM and presents the signal as an immobilized cue to cells. Heparin sequesters growth factors including VEGF [85], sonic hedgehog [86], and FGFs [87], which are growth factors commonly used in PSC differentiation protocols. While these growth factors have successfully modulated PSC fate as soluble additions, in some circumstances, certain growth factors possess increased potency when immobilized [35, 88]. One method to mimic ECM sequestration is to incorporate heparin within the scaffold to facilitate natural growth factor sequestration [7, 89]. Another method to mimic ECM sequestration is to directly immobilize specific growth factors to the scaffold backbone. These biochemical additions to scaffolds can further our control and better mimic the native environment for differentiation of PSCs.

### 4.3. Immobilized Biochemical Cues for Mimicking Cell-Cell Signaling

Cell-cell membrane bound ligand receptor signaling is another example of an important cue to stem cells that is not readily manipulated with standard culture practices. With the addition of biomaterials, researchers can now emulate these interactions via immobilization of proteins to the scaffold. Such techniques have been employed to mimic cell-cell signals for neural [12, 81, 83] and vascular [38, 90, 91] differentiation targeting pathways that include ephrin-Eph [83], sonic hedgehog [81], and Notch [38, 90]. Both ligand and receptor are expressed on cell membranes and require cell-cell contact to initiate signaling; soluble ligands elicit limited to no cellular response. For example, Notch signaling has been targeted for vascular differentiation protocols by surface immobilizing Notch ligands Jagged1 or Dll1. Immobilized ligands but not soluble ligands were successful in activating the Notch pathway and increasing vascular differentiation [90].

Some biological signals, such as ephrins-Eph and sonic hedgehog signalings, require receptor clustering or multivalent interactions to activate downstream signaling pathways. Conway et al. found ephrinB2 signaling potency with respect to enhancement of PSC neural differentiation was increased as a function of the number of ephrinB2 proteins immobilized along a polymer chain backbone [83]. In our lab, we demonstrated how ephrinB2/EphB4-immobilized signaling can be used to promote the distinction of arterial venous differentiation of PSCs [39]. Similar to Conway et al., we observed that immobilization of ephrinB2/EphB4 proteins was required to achieve an effect, highlighting the importance biomaterials can play on controlling stem cell fate.

### 4.4. Immobilized Biochemical Cues for Creating Spatial Patterns

Researchers have also been motivated to immobilize growth factors to biomaterials with the purpose of creating spatial patterns. There are many examples throughout tissue development and maturation of biochemical spatial patterns including retina patterning [92] and spinal cord development [93]. Additionally, gradient patterns are a great method for high throughput testing for optimal concentrations for a particular stem cell fate. For example, a N-cadherin peptide gradient patterned on a PEG hydrogel substrate was developed to identify the optimal concentration for neural differentiation [12]. Similarly, a continuous gradient of laminin peptide, IKVAV, was fabricated to optimize adhesive conditions for neural differentiation [13]. These approaches allow testing a large range of concentrations to pinpoint optimal differentiation conditions. Discrete patterns have also been utilized within PSC differentiation schemes. For example, researchers have surface patterned VEGF and observed site-specific differentiation [34]. Specifically, they found that locations with immobilized VEGF yielded higher endothelial commitment compared to areas without VEGF which favored vascular smooth muscle-like cell fate [34]. These spatial specific patterns illustrate the potential for a biomaterial platform to accelerate protocol optimization and to explore spatial specific signals as they relate to development and cell fate.

## 5. Biomaterials for Overcoming PSC Translational Challenges

While there is a lot of excitement surrounding PSCs, there are certain roadblocks that limit these translations of these technologies into the clinics. Biomaterial research has begun to address some of these challenges. First, there is a need for an easily configurable culture system that permits long-term, high-volume PSC maintenance. One group designed a macrofibrous synthetic platform consisting of electrospun gelatin nanofibers for PSC cultures. This design showed it could support large-scale (55 ml) and long-term culture (2 months) of human PSCs [23]. Another scaffold design to address scalability issues was developed: thermoresponsive 3D PNIPAAm-PEG hydrogel. This culture system was able to retain high pluripotency purity with a 20-fold expansion rate for 5 d passage [42]. The same 3D scaffold has also been explored as a culture system for the differentiation of neurons [11] and oligodendrocytes [41] for cell therapy applications. Again, they found their biomaterial platform supported scaling up cultures and achieved high yields of desired cell fate.

Another challenge current PSC differentiation protocols face is the reliance on soluble growth factors. In 2D differentiation cultures, the required amount of growth factors can become expensive and unrealistic for high volume systems. Alternatively, a more economical approach is to immobilize signals within a 3D biomaterial or encapsulate growth factors for localized release [94, 95]. In one study, growth factor encapsulation within 3D, degradable polymeric microparticles led to a 10-fold reduction in total growth factor needed to stimulate comparable gene expression to soluble treatment in 2D cultures [95].

PSC applications in high throughput drug screening and *in vitro* tissue models are limited due to PSC sensitivity to processing techniques. Retaining PSC pluripotency and survival during bioprinting is a concern. Bioinks are being developed to support PSC health and limit undesired spontaneous differentiation during the printing process [96, 97].

## 6. Summary

**Figure 5:**
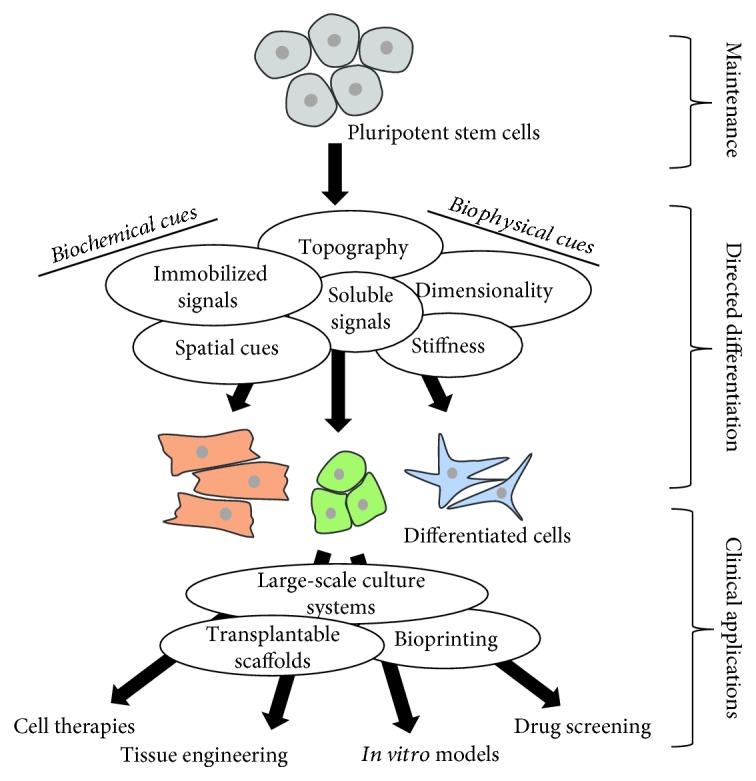
Summary of PSC technology, methods for directing differentiation, and potential avenues for biomaterial contribution to translation into regenerative medicine and personalized healthcare.

In summary, biomaterial engineering for PSCs is an expanding field with immense potential for furthering our control over stem cell fate and translating PSC technologies into clinical applications.  Figure 5 illustrates how biomaterials can be used for directing differentiation and downstream applications of PSC-derived populations. Biomaterials provide new methodologies for influencing stem cell fate via engineered microenvironments with both biophysical and biochemical cues. These environmental signals can increase differentiation efficiency and improve functionality of PSC-derived populations as well as be an alternative culture system to facilitate the translation of PSC technologies into clinically viable therapies.

## Figures and Tables

**Table 1:** Biomaterial scaffolds used to create microenvironments for influencing PSC fate.

	Material	Crosslink chemistry	Effect on cell lineages
Natural	Collagen I	pH-sensitive crosslinking Temperature Photopolymerization	Pluripotency [21] Neural [3]
	Gelatin	Photopolymerization Temperature Passive coating	Osteogenic [1, 22] Chondrogenesis [9] Pluripotency [23] Pancreatic [10] Retinal [24]
	Fibrin	Enzymatic crosslinking	Endoderm [25] Neural [26, 27] Osteogenic [28]
	Hyaluronic acid (HA)	Photopolymerization	Pluripotency [29, 30] Neural [2, 3] Retinal [24]
	Cell-derived ECMs/Matrigel	Thermosensitive crosslinking	Pluripotency [31, 32] Vascular [15, 33] Neural [4, 32]
	Chitosan	Photopolymerization	Vascular [34] Osteogenic [1]
	Agarose	Thermosensitive hydrogel	Vascular [35]
	Dextran	Photopolymerization	Vascular [20]
	Alginate	CaCl_2_ crosslinking Photopolymerization	Neural [2] Retinal [16] Primordial germ cells [36] Osteogenic [28]
	Chondroitin sulfate	Glutaraldehyde crosslinking	Retinal [24]
	Hydroxyapatite; calcium phosphate	Glutaraldehyde crosslinking Freeze-dried	Osteogenic [1, 28]
	RADA16-I/II	Self-assembling peptides	Vascular (cardiac) [37, 38]
Synthetic	Poly(ethylene glycol) (PEG)	Photopolymerization	Neural [11–14] Vascular [15, 39]
	Poly(L-lysine) (PLL)	Polyelectrolyte films EDC crosslinking	Germ lineages [29]
	Polycaprolactone (PCL)	Electrospinning	Neural [4, 5] Osteogenic [6] Chondrogenic [9]
	Polylactic acid (PLA)	Electrospinning	Definitive endoderm [40]
	Poly(lactic-co-glycolic acid) (PLGA)	Electrospinning Freeze-dried	Neural [7, 8] Pancreatic [10]
	N-Isopropylacrylamide gels (NIPAAm)	Thermosensitive hydrogel	Neural [11, 41] Pluripotency [42]

**Table 2:** ECM-derived proteins/peptides used within PSC culture systems to control cell fate.

	ECM protein/peptide	Immobilization chemistry	Lineage
Proteins	Vitronectin	Polydopamine immobilization	Pluripotency [18, 30, 43] Reprogramming [18]
	Collagen IV	EDC conjugation	Vascular [34] Primordial germ cells [36]
	Heparin	EDC conjugation	Pluripotency [21] Neural [7]
Peptides	Laminin peptide (IKVAV)	Photochemistry	Neural [13, 14]
	Laminin peptide (YIGSR)	Photochemistry	Neural [14]
	Laminin 5 peptide (Ln5-P4) PPFLMLLKGSTR	EDC conjugation	Neural [3]
	Fibronectin peptide (RGD)	Photochemistry EDC conjugation	Neural [3, 14] Retinal [16] Hepatocytic [17] Pluripotency [18] Osteogenic [19] Vascular [20]
	Vitronectin peptide GGPQVTRGDVFTMP	EDC conjugation	Hepatocytic [17] Pluripotency [43–45] Osteogenic [30, 45]
	MMP degradable peptide GCRDV¯P¯M¯S¯↓¯M¯R¯G¯G¯DRCG	Photochemistry	Pluripotency [14]

**Table 3:** Biomaterials and controlled stiffness to modulate PSC fate.

	Material	Variables to modulate stiffness	Stiffness range	Lineage studied
Natural	Fibrin	Precursor concentration	Pa	Endoderm [25]
	Fibroblast-derived matrix	Crosslinking concentration	Pa–KPa	Pluripotency [31]
	Alginate	Crosslinking concentration	KPa	Pancreatic [72]
Synthetic	PDMS	Precursor concentration	KPa–MPa	Endo-meso-ectoderm [59] Pluripotency [58] Vascular [60]
	Polyacrylamide	Precursor concentration	Pa–KPa	Neural [67] Cellular reprogramming [66]
	PEG hydrogel	Precursor concentration	KPa–MPa	Vascular [39]

## References

[1] Ji J., Tong X., Huang X. (2015). Sphere-shaped nano-hydroxyapatite/chitosan/gelatin 3D porous scaffolds increase proliferation and osteogenic differentiation of human induced pluripotent stem cells from gingival fibroblasts. *Biomedical Materials*.

[2] Bozza A., Coates E. E., Incitti T. (2014). Neural differentiation of pluripotent cells in 3D alginate-based cultures. *Biomaterials*.

[3] Kuo Y.-C., Hsueh C.-H. (2017). Neuronal production from induced pluripotent stem cells in self-assembled collagen-hyaluronic acid-alginate microgel scaffolds with grafted GRGDSP/Ln5-P4. *Materials Science and Engineering: C*.

[4] Terraf P., Babaloo H., Kouhsari S. M. (2017). Directed differentiation of dopamine-secreting cells from Nurr1/GPX1 expressing murine embryonic stem cells cultured on Matrigel-coated PCL scaffolds. *Molecular Neurobiology*.

[5] Mohtaram N. K., Ko J., King C. (2015). Electrospun biomaterial scaffolds with varied topographies for neuronal differentiation of human-induced pluripotent stem cells. *Journal of Biomedial Materials Research Part A*.

[6] Deng Y., Yang Y., Wei S. (2017). Peptide-decorated nanofibrous niche augments in vitro directed osteogenic conversion of human pluripotent stem cells. *Biomacromolecules*.

[7] Meade K. A., White K. J., Pickford C. E. (2013). Immobilization of heparan sulfate on electrospun meshes to support embryonic stem cell culture and differentiation. *The Journal of Biological Chemistry*.

[8] Sperling L. E., Reis K. P., Pozzobon L. G., Girardi C. S., Pranke P. (2017). Influence of random and oriented electrospun fibrous poly (lactic-co-glycolic acid) scaffolds on neural differentiation of mouse embryonic stem cells. *Journal of Biomedical Materials Research. Part A*.

[9] Liu J., Nie H., Xu Z. (2014). The effect of 3D nanofibrous scaffolds on the chondrogenesis of induced pluripotent stem cells and their application in restoration of cartilage defects. *PLoS One*.

[10] Kuo Y.-C., Liu Y.-C., Rajesh R. (2017). Pancreatic differentiation of induced pluripotent stem cells in activin A-grafted gelatin-poly (lactide-*co*-glycolide) nanoparticle scaffolds with induction of LY294002 and retinoic acid. *Materials Science and Engineering: C*.

[11] Adil M. M., Rodrigues G. M. C., Kulkarni R. U. (2017). Efficient generation of hPSC-derived midbrain dopaminergic neurons in a fully defined, scalable, 3D biomaterial platform. *Scientific Reports*.

[12] Lim H. J., Mosley M. C., Kurosu Y., Smith Callahan L. A. (2017). Concentration dependent survival and neural differentiation of murine embryonic stem cells cultured on polyethylene glycol dimethacrylate hydrogels possessing a continuous concentration gradient of n-cadherin derived peptide His-Ala-Val-Asp-Lle. *Acta Biomaterialia*.

[13] Yang Y.-H., Khan Z., Ma C., Lim H. J., Smith Callahan L. A. (2015). Optimization of adhesive conditions for neural differentiation of murine embryonic stem cells using hydrogels functionalized with continuous Ile-Lys-Val-Ala-Val concentration gradients. *Acta Biomaterialia*.

[14] Ovadia E. M., Colby D. W., Kloxin A. M. (2018). Designing well-defined photopolymerized synthetic matrices for three-dimensional culture and differentiation of induced pluripotent stem cells. *Biomaterials Science*.

[15] Zhang J., Schwartz M. P., Hou Z. (2017). A genome-wide analysis of human pluripotent stem cell-derived endothelial cells in 2D or 3D culture. *Stem Cell Reports*.

[16] Hunt N. C., Hallam D., Karimi A. (2017). 3D culture of human pluripotent stem cells in RGD-alginate hydrogel improves retinal tissue development. *Acta Biomaterialia*.

[17] Nagaoka M., Kobayashi M., Kawai C., Mallanna S. K., Duncan S. A. (2015). Design of a vitronectin-based recombinant protein as a defined substrate for differentiation of human pluripotent stem cells into hepatocyte-like cells. *PLoS One*.

[18] Zhou P., Wu F., Zhou T. (2016). Simple and versatile synthetic polydopamine-based surface supports reprogramming of human somatic cells and long-term self-renewal of human pluripotent stem cells under defined conditions. *Biomaterials*.

[19] Wang M., Deng Y., Zhou P. (2015). In vitro culture and directed osteogenic differentiation of human pluripotent stem cells on peptides-decorated two-dimensional microenvironment. *Applied Materials & Interfaces*.

[20] Ferreira L. S., Gerecht S., Fuller J., Shieh H. F., Vunjak-Novakovic G., Langer R. (2007). Bioactive hydrogel scaffolds for controllable vascular differentiation of human embryonic stem cells. *Biomaterials*.

[21] Lee M., Kim Y., Ryu J. H., Kim K., Han Y. M., Lee H. (2016). Long-term, feeder-free maintenance of human embryonic stem cells by mussel-inspired adhesive heparin and collagen type I. *Acta Biomaterialia*.

[22] Kang H., Shih Y. R. V., Hwang Y. (2014). Mineralized gelatin methacrylate-based matrices induce osteogenic differentiation of human induced pluripotent stem cells. *Acta Biomaterialia*.

[23] Liu L., Kamei K. I., Yoshioka M. (2017). Nano-on-micro fibrous extracellular matrices for scalable expansion of human ES/iPS cells. *Biomaterials*.

[24] Singh D., Wang S. B., Xia T. (2018). A biodegradable scaffold enhances differentiation of embryonic stem cells into a thick sheet of retinal cells. *Biomaterials*.

[25] Jaramillo M., Singh S. S., Velankar S., Kumta P. N., Banerjee I. (2015). Inducing endoderm differentiation by modulating mechanical properties of soft substrates. *Journal of Tissue Engineering and Regenerative Medicine*.

[26] McCreedy D. A., Wilems T. S., Xu H. (2014). Survival, differentiation, and migration of high-purity mouse embryonic stem cell-derived progenitor motor neurons in fibrin scaffolds after sub-acute spinal cord injury. *Biomaterials Science*.

[27] Montgomery A., Wong A., Gabers N., Willerth S. M. (2015). Engineering personalized neural tissue by combining induced pluripotent stem cells with fibrin scaffolds. *Biomaterials Science*.

[28] Wang L., Zhang C., Li C. (2016). Injectable calcium phosphate with hydrogel fibers encapsulating induced pluripotent, dental pulp and bone marrow stem cells for bone repair. *Materials Science and Engineering: C*.

[29] Blin G., Lablack N., Louis-Tisserand M., Nicolas C., Picart C., Pucéat M. (2010). Nano-scale control of cellular environment to drive embryonic stem cells selfrenewal and fate. *Biomaterials*.

[30] Deng Y., Zhang X., Zhao Y. (2014). Peptide-decorated polyvinyl alcohol/hyaluronan nanofibers for human induced pluripotent stem cell culture. *Carbohydrate Polymers*.

[31] Kim I. G., Gil C. H., Seo J. (2018). Mechanotransduction of human pluripotent stem cells cultivated on tunable cell-derived extracellular matrix. *Biomaterials*.

[32] Yan Y., Martin L. M., Bosco D. B. (2015). Differential effects of acellular embryonic matrices on pluripotent stem cell expansion and neural differentiation. *Biomaterials*.

[33] Feaster T. K., Cadar A. G., Wang L. (2015). Matrigel mattress. *Circulation Research*.

[34] Chiang C. K., Chowdhury M. F., Iyer R. K., Stanford W. L., Radisic M. (2010). Engineering surfaces for site-specific vascular differentiation of mouse embryonic stem cells. *Acta Biomaterialia*.

[35] Rahman N., Purpura K. A., Wylie R. G., Zandstra P. W., Shoichet M. S. (2010). The use of vascular endothelial growth factor functionalized agarose to guide pluripotent stem cell aggregates toward blood progenitor cells. *Biomaterials*.

[36] Mansouri V., Salehi M., Omrani M. ., Niknam Z., Ardeshirylajimi A. (2017). Collagen-alginate microspheres as a 3D culture system for mouse embryonic stem cells differentiation to primordial germ cells. *Biologicals*.

[37] Puig-Sanvicens V. A. C., Semino C. E., Zur Nieden N. I. (2015). Cardiac differentiation potential of human induced pluripotent stem cells in a 3D self-assembling peptide scaffold. *Differentiation*.

[38] Boopathy A. V., Che P. L., Somasuntharam I. (2014). The modulation of cardiac progenitor cell function by hydrogel dependent Notch1 activation. *Biomaterials*.

[39] Dorsey T. B., Kim D., Grath A., James D., Dai G. (2018). Multivalent biomaterial platform to control the distinct arterial venous differentiation of pluripotent stem cells. *Biomaterials*.

[40] Hoveizi E., Nabiuni M., Parivar K., Ai J., Massumi M. (2014). Definitive endoderm differentiation of human-induced pluripotent stem cells using signaling molecules and IDE1 in three-dimensional polymer scaffold. *Journal of Biomedical Materials Research Part A*.

[41] Rodrigues G. M. C., Gaj T., Adil M. M. (2017). Defined and scalable differentiation of human oligodendrocyte precursors from pluripotent stem cells in a 3D culture system. *Stem Cell Reports*.

[42] Lei Y., Schaffer D. V. (2013). A fully defined and scalable 3D culture system for human pluripotent stem cell expansion and differentiation. *PNAS*.

[43] Deng Y., Zhang X., Zhao X. (2013). Long-term self-renewal of human pluripotent stem cells on peptide-decorated poly(OEGMA-co-HEMA) brushes under fully defined conditions. *Acta Biomaterialia*.

[44] Chen Y.-M., Chen L.-H., Li M.-P. (2017). Xeno-free culture of human pluripotent stem cells on oligopeptide-grafted hydrogels with various molecular designs. *Scientific Reports*.

[45] Deng Y., Wei S., Yang L., Yang W., Dargusch M. S., Chen Z. G. (2018). A novel hydrogel surface grafted with dual functional peptides for sustaining long-term self-renewal of human induced pluripotent stem cells and manipulating their osteoblastic maturation. *Advanced Functional Materials*.

[46] Shijun X., Junsheng M., Jianqun Z., Ping B. (2015). *In vitro* three-dimensional coculturing poly3-hydroxybutyrate-*co*-3-hydroxyhexanoate with mouse-induced pluripotent stem cells for myocardial patch application. *Journal of Biomaterials Applications*.

[47] Richardson T., Kumta P. N., Banerjee I. (2014). Alginate encapsulation of human embryonic stem cells to enhance directed differentiation to pancreatic islet-like cells. *Tissue Engineering. Part A*.

[48] Baharvand H., Hashemi S. M., Kazemi Ashtiani S., Farrokhi A. (2006). Differentiation of human embryonic stem cells into hepatocytes in 2D and 3D culture systems in vitro. *The International Journal of Developmental Biology*.

[49] Shao Y., Taniguchi K., Gurdziel K. (2017). Self-organized amniogenesis by human pluripotent stem cells in a biomimetic implantation-like niche. *Nature Materials*.

[50] Heydarkhan-Hagvall S., Gluck J. M., Delman C. (2012). The effect of vitronectin on the differentiation of embryonic stem cells in a 3D culture system. *Biomaterials*.

[51] Butcher D. T., Alliston T., Weaver V. M. (2009). A tense situation: forcing tumour progression. *Nature Reviews. Cancer*.

[52] Levy-mishali M., Zoldan J., Levenberg S. (2009). Effect of scaffold stiffness on myoblast differentiation. *Tissue Engineering. Part A*.

[53] Yuan H., Zhou Y., Lee M. S., Zhang Y., Li W. J. (2016). A newly identified mechanism involved in regulation of human mesenchymal stem cells by fibrous substrate stiffness. *Acta Biomaterialia*.

[54] Hasan A., Soliman S., el Hajj F., Tseng Y. T., Yalcin H. C., Marei H. E. (2018). Fabrication and in vitro characterization of a tissue engineered PCL-PLLA heart valve. *Scientific Reports*.

[55] Ju D., Han L., Li F., Chen S., Dong L. (2013). Crystallization, mechanical properties, and enzymatic degradation of biodegradable poly(*ε*-caprolactone) composites with poly(lactic acid) fibers. *Polymer Composites*.

[56] Blakney A. K., Swartzlander M. D., Bryant S. J. (2012). The effects of substrate stiffness on the *in vitro* activation of macrophases and *in vivo* host response to poly(ethylene glycol)-based hydrogels. *Journal of Biomedial Materials Research Part A*.

[57] Dorsey T. B., Grath A., Wang A., Xu C., Hong Y., Dai G. (2018). Evaluation of photochemistry reaction kinetics to pattern bioactive proteins on hydrogels for biological applications. *Bioactive Materials*.

[58] Fu J., Chuah Y. J., Ang W. T., Zheng N., Wang D. A. (2017). Optimization of a polydopamine (PD)-based coating method and polydimethylsiloxane (PDMS) substrates for improved mouse embryonic stem cell (ESC) pluripotency maintenance and cardiac differentiation. *Biomaterials Science*.

[59] Evans N. D., Minelli C., Gentleman E. (2009). Substrate stiffness affects early differentiation events in embryonic stem cells. *European Cells and Materials*.

[60] Smith Q., Chan X. Y., Carmo A. M., Trempel M., Saunders M., Gerecht S. (2017). Compliant substratum guides endothelial commitment from human pluripotent stem cells. *Science Advances*.

[61] Li X., Zhang J., Kawazoe N., Chen G. (2017). Fabrication of highly crosslinked gelatin hydrogel and its influence on chondrocyte proliferation and phenotype. *Polymers*.

[62] Zuidema J. M., Rivet C. J., Gilbert R. J., Morrison F. A. (2014). A protocol for rheological characterization of hydrogels for tissue engineering strategies. *Journal of Biomedical Materials Research Part B: Applied Biomaterials*.

[63] Balgude A. P., Yu X., Szymanski A., Bellamkonda R. V. (2001). Agarose gel stiffness determines rate of DRG neurite extension in 3D cultures. *Biomaterials*.

[64] Duong H., Wu B., Tawil B. (2009). Modulation of 3D fibrin matrix stiffness by intrinsic fibrinogen–thrombin compositions and by extrinsic cellular activity. *Tissue Engineering. Part A*.

[65] Burdick J. A., Vunjak-novakovic G. (2009). Engineered microenvironments for controlled stem cell differentiation. *Tissue Engineering. Part A*.

[66] Choi B., Park K. S., Kim J. H. (2016). Stiffness of hydrogels regulates cellular reprogramming efficiency through mesenchymal-to-epithelial transition and stemness markers. *Macromolecular Bioscience*.

[67] Keung A. J., Asuri P., Kumar S., Schaffer D. V. (2012). Soft microenvironments promote the early neurogenic differentiation but not self-renewal of human pluripotent stem cells. *Integrative Biology*.

[68] Hutmacher D. W., Schantz T., Zein I., Ng K. W., Teoh S. H., Tan K. C. (2001). Mechanical properties and cell cultural response of polycaprolactone scaffolds designed and fabricated via fused deposition modeling. *Journal of Biomedical Materials Research*.

[69] Denisin A. K., Pruitt B. L. (2016). Tuning the range of polyacrylamide gel stiffness for mechanobiology applications. *Applied Materials & Interfaces*.

[70] Li Z., Guo X., Palmer A. F., Das H., Guan J. (2012). High-efficiency matrix modulus-induced cardiac differentiation of human mesenchymal stem cells inside a thermosensitive hydrogel. *Acta Biomaterialia*.

[71] Price A. J., Huang E. Y., Sebastiano V., Dunn A. R. (2017). A semi-interpenetrating network of polyacrylamide and recombinant basement membrane allows pluripotent cell culture in a soft, ligand-rich microenvironment. *Biomaterials*.

[72] Richardson T., Barner S., Candiello J., Kumta P. N., Banerjee I. (2016). Capsule stiffness regulates the efficiency of pancreatic differentiation of human embryonic stem cells. *Acta Biomaterialia*.

[73] Cooper A., Leung M., Zhang M. (2012). Polymeric fibrous matrices for substrate-mediated human embryonic stem cell lineage differentiation. *Macromolecular Bioscience*.

[74] Zhang C., Yuan H., Liu H. (2015). Well-aligned chitosan-based ultrafine fibers committed teno-lineage differentiation of human induced pluripotent stem cells for Achilles tendon regeneration. *Biomaterials*.

[75] Li K., Zhong X., Yang S. (2017). HiPSC-derived retinal ganglion cells grow dendritic arbors and functional axons on a tissue-engineered scaffold. *Acta Biomaterialia*.

[76] Ji J., Tong X., Huang X., Zhang J., Qin H., Hu Q. (2016). Patient-derived human induced pluripotent stem cells from gingival fibroblasts composited with defined nanohydroxyapatite/chitosan/gelatin porous scaffolds as potential bone graft substitutes. *Stem Cells Translational Medicine*.

[77] Abagnale G., Sechi A., Steger M. (2017). Surface topography guides morphology and spatial patterning of induced pluripotent stem cell colonies. *Stem Cell Reports*.

[78] Lee M. R., Kwon K. W., Jung H. (2010). Direct differentiation of human embryonic stem cells into selective neurons on nanoscale ridge/groove pattern arrays. *Biomaterials*.

[79] Lyu Z., Wang H., Wang Y. (2014). Maintaining the pluripotency of mouse embryonic stem cells on gold nanoparticle layers with nanoscale but not microscale surface roughness. *Nanoscale*.

[80] Alberti K., Davey R. E., Onishi K. (2008). Functional immobilization of signaling proteins enables control of stem cell fate. *Nature Methods*.

[81] Vazin T., Ashton R. S., Conway A. (2014). The effect of multivalent sonic hedgehog on differentiation of human embryonic stem cells into dopaminergic and GABAergic neurons. *Biomaterials*.

[82] Conway A., Spelke D. P., Schaffer D. V. (2014). Conjugation of proteins to polymer chains to create multivalent molecules. *Methods in Molecular Biology*.

[83] Conway A., Vazin T., Spelke D. P. (2013). Multivalent ligands control stem cell behaviour in vitro and in vivo. *Nature Nanotechnology*.

[84] Çetinkaya G., Türkoğlu H., Arat S. (2007). LIF-immobilized nonwoven polyester fabrics for cultivation of murine embryonic stem cells. *Journal of Biomedical Materials Research Part A*.

[85] Krilleke D., DeErkenez A., Schubert W. (2007). Molecular mapping and functional characterization of the VEGF164 heparin-binding domain. *The Journal of Biological Chemistry*.

[86] McLellan J. S., Yao S., Zheng X. (2006). Structure of a heparin-dependent complex of hedgehog and ihog. *Proceedings of the National Academy of Sciences of the United States of America*.

[87] Lippmann E. S., Azarin S. M., Kay J. E. (2012). Derivation of blood-brain barrier endothelial cells from human pluripotent stem cells. *Nature Biotechnology*.

[88] Minato A., Ise H., Goto M., Akaike T. (2012). Cardiac differentiation of embryonic stem cells by substrate immobilization of insulin-like growth factor binding protein 4 with elastin-like polypeptides. *Biomaterials*.

[89] Adil M. M., Vazin T., Ananthanarayanan B. (2017). Engineered hydrogels increase the post-transplantation survival of encapsulated hESC-derived midbrain dopaminergic neurons. *Biomaterials*.

[90] Lee J. B., Werbowetski-Ogilvie T. E., Lee J. H. (2013). Notch-HES1 signaling axis controls hemato-endothelial fate decisions of human embryonic and induced pluripotent stem cells. *Blood*.

[91] Tung J. C., Paige S. L., Ratner B. D., Murry C. E., Giachelli C. M. (2014). Engineered biomaterials control differentiation and proliferation of human-embryonic-stem-cell-derived cardiomyocytes via timed Notch activation. *Stem Cell Reports*.

[92] Sloan T. F. W., Qasaimeh M. A., Juncker D., Yam P. T., Charron F. (2015). Integration of shallow gradients of Shh and Netrin-1 guides commissural axons. *PLoS Biology*.

[93] Avilés E. C., Wilson N. H., Stoeckli E. T. (2013). Sonic hedgehog and Wnt: antagonists in morphogenesis but collaborators in axon guidance. *Frontiers in Cellular Neuroscience*.

[94] Memanishvili T., Kupatadze N., Tugushi D. (2016). Generation of cortical neurons from human induced-pluripotent stem cells by biodegradable polymeric microspheres loaded with priming factors. *Biomedical Materials*.

[95] Heidariyan Z., Ghanian M. H., Ashjari M. (2018). Efficient and cost-effective generation of hepatocyte-like cells through microparticle-mediated delivery of growth factors in a 3D culture of human pluripotent stem cells. *Biomaterials*.

[96] Tronser T., Popova A. A., Jaggy M., Bastmeyer M., Levkin P. A. (2017). Droplet microarray based on patterned superhydrophobic surfaces prevents stem cell differentiation and enables high-throughput stem cell screening. *Advanced Healthcare Materials*.

[97] Koch L., Deiwick A., Franke A. (2018). Laser bioprinting of human induced pluripotent stem cells—the effect of printing and biomaterials on cell survival, pluripotency, and differentiation. *Biofabrication*.

